# FTO3/408: Using Java for Real-Time Image Fusion on Open MR Systems

**DOI:** 10.2196/jmir.1.suppl1.e28

**Published:** 1999-09-19

**Authors:** C Burger, P Rudnicki, M Grodzki, K Mikolajczyk

**Affiliations:** ^1^Universitätsspital Zürich, ZürichSwitzerland

**Keywords:** PET, Kinetic Modeling

## Abstract

**Introduction:**

With interventional magnetic resonance imaging (MRI) a new era of non-invasive procedures has begun. MR-compliant tools allow to perform biopsies and surgery during ongoing MR-imaging. However, the interventional images are typically of limited quality and do not show functional information. They are thus difficult to interpret by the surgeon. The aim of the current project was to improve the perception of the operational situation by combining the interventional images with additional information from prior imaging studies.

**Methods:**

A software environment has been developed which is able to communicate with an open MR scanner (GE 0.5T Signa SP) and which presents an advanced image fusion environment to the surgeon. Prior imaging studies of the patient are initially loaded, e.g. a FDG-PET study and/or a high-resolution MR acquired on a high-field system. After the patient has been prepared for the operation, a volumetric MR image set is acquired to which the pre-loaded image sets are geometrically aligned. During the operation single-slice images are acquired at arbitrary orientations according to the surgeon's commands. Each one is immediately retrieved by the fusion system together with its slice definition parameters, the matched slice of one of the reference studies is generated and an image is displayed which shows the fused information. The system is based on a client-server architecture and exchanges data over TCP sockets. Except for the server running on GE's MR realtime-workstation the entire program was written in Java 2.

**Results:**

The real-time fusion software has been tested in several interventions. Although being a Java application it has proven to run reliably and delivers the fused images with a delay of only about 0.5 seconds on a 400 MHz PII system. First experiences suggest that the joint presentation of supplementary information together with the real-time images in fused images makes MR-guided interventions more easy and accurate.

**Discussion:**

Image fusion allows the surgeon to exploit high-resolution anatomical information and/or functional PET information in addition to the low-quality interventional MR images. This is particularly useful when deciding how to enter the skull for optimally approaching the target. During the intervention itself, the brain may undergo geometrical changes, eg. as a consequence of a craniotomy or cyst drainage. After such an event, prior images do not match the current situation and must not be used for fusion purposes any more. However, the fusion system may still support the interventionalist for important decisions: A high-quality volumetric MR scan can be acquired during a short break and be used as the new reference image during the next phase of the intervention.

Acknowledgement:

This work has been supported by the EMDO Foundation.

